# A Combination of Silicone Sheets for the Treatment of Adhesions in the Nasal Cavity

**DOI:** 10.7759/cureus.75755

**Published:** 2024-12-15

**Authors:** Kazuhiro Nomura, Tomotaka Hemmi, Mitsuru Sugawara, Risako Kakuta

**Affiliations:** 1 Otolaryngology, Tohoku Kosai Hospital, Sendai, JPN; 2 Otolaryngology - Head and Neck Surgery, Tohoku University Graduate School of Medicine, Sendai, JPN

**Keywords:** endoscopic sinus surgery, nasal septum, surgery, synechia, treatment

## Abstract

Nasal adhesions, or synechiae, commonly occur following surgical procedures, resulting in nasal airway obstruction and patient discomfort. While various packing materials are available to prevent adhesion formation post-surgery, there is limited guidance on effectively dividing existing adhesions and determining the optimal packing materials to maintain separation afterward. We treated a 59-year-old man with severe adhesions in the anterior nasal cavity. After dividing the adhesions, we placed a combination of silicone sheets to cover the nasal septum, inferior turbinate, and inferior meatus. The airway remained open for one year following the procedure. Silicone is so far the best material to cover the raw surface of the nasal cavity. Being chemically inert, it does not easily interact with bodily tissues, resulting in minimal inflammatory responses. This low level of tissue reaction decreases the risk of significant inflammation or immune responses and helps to prevent tissue assimilation or excessive scarring around the material. Here, we present the treatment history and our novel technique utilizing silicone sheets.

## Introduction

Nasal adhesions or synechiae commonly occur after surgical procedures, leading to nasal airway obstruction and patient discomfort, and can sometimes result in physiological dysfunctions, including obstructive sleep apnea [1-3]. One study showed that a total of 286 patients underwent endoscopic sinus surgery, with 55 (19.2%) developing synechiae in the follow-up period [3]. Various packing materials are available to prevent adhesion formation post-surgery. However, when adhesions do form, there is limited guidance on how to effectively divide them and which packing materials are optimal for maintaining separation post-division. No studies have examined the division of adhesions, and no quantitative results are available.

Adhesions in the anterior part of the nasal cavity significantly alter local airflow and reduce mucosal cooling in and around the area of the adhesions [4]. Any nasal surgery can lead to adhesions as a result of abrasions to the mucosal surface in narrow spaces. Endoscopic sinus surgery, inferior turbinectomy, and septoplasty are common procedures that may cause adhesions. These adhesions typically form between the nasal septum and the inferior turbinate or at the inferior meatus. Pre-operative and post-operative infections might also contribute to the development of adhesions, but no reports specifically address this. While the division of adhesions may seem simple, reformation often occurs within weeks after the initial procedure. We successfully treated a large adhesion involving both the anterior nasal cavity between the nasal septum and the inferior turbinate, as well as the anterior entrance of the inferior meatus, following a second attempt after an initial failure. Here, we present the technique and the packing device used in this treatment.

## Case presentation

**Figure 1:**
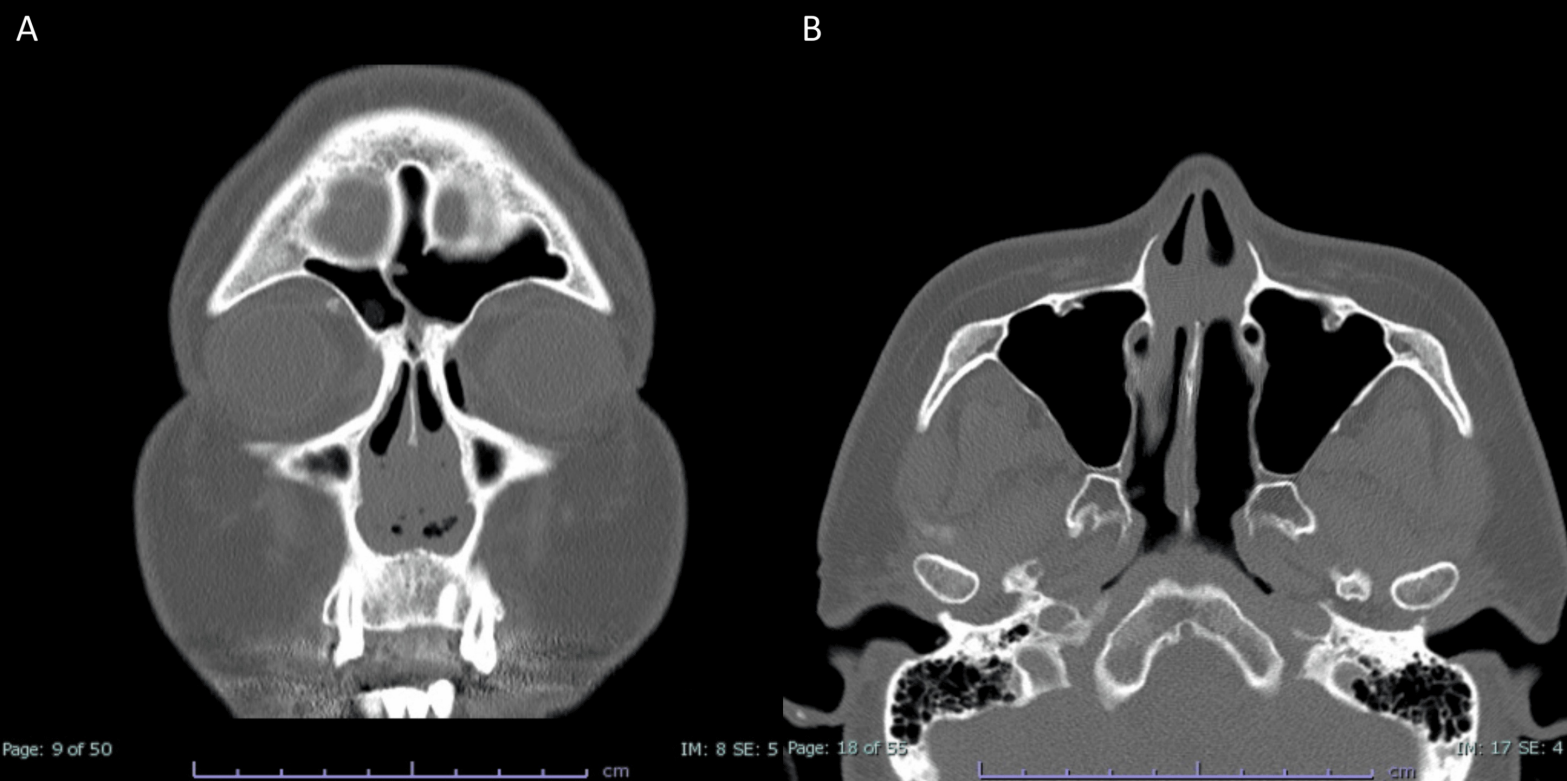
CT scan of the nasal cavity at the initial visit. A) Coronal view showing bilateral nostril obstruction due to adhesions. B) Axial image indicating that the adhesions are confined to the anterior part of the nasal cavity.

A 59-year-old man was referred to our hospital for adhesion of the nasal cavity. He had no history of nasal trauma or surgery. He had not been in an environment that could have caused the adhesions. Additionally, there was no history of cocaine use or substance use disorder. He had untreated hypertension. He did not have any psychiatric issues. On the initial visit, extensive adhesions were observed in both nostrils, and CT imaging confirmed that these were limited to the anterior nasal cavity (Figure 1).

**Figure 2:**
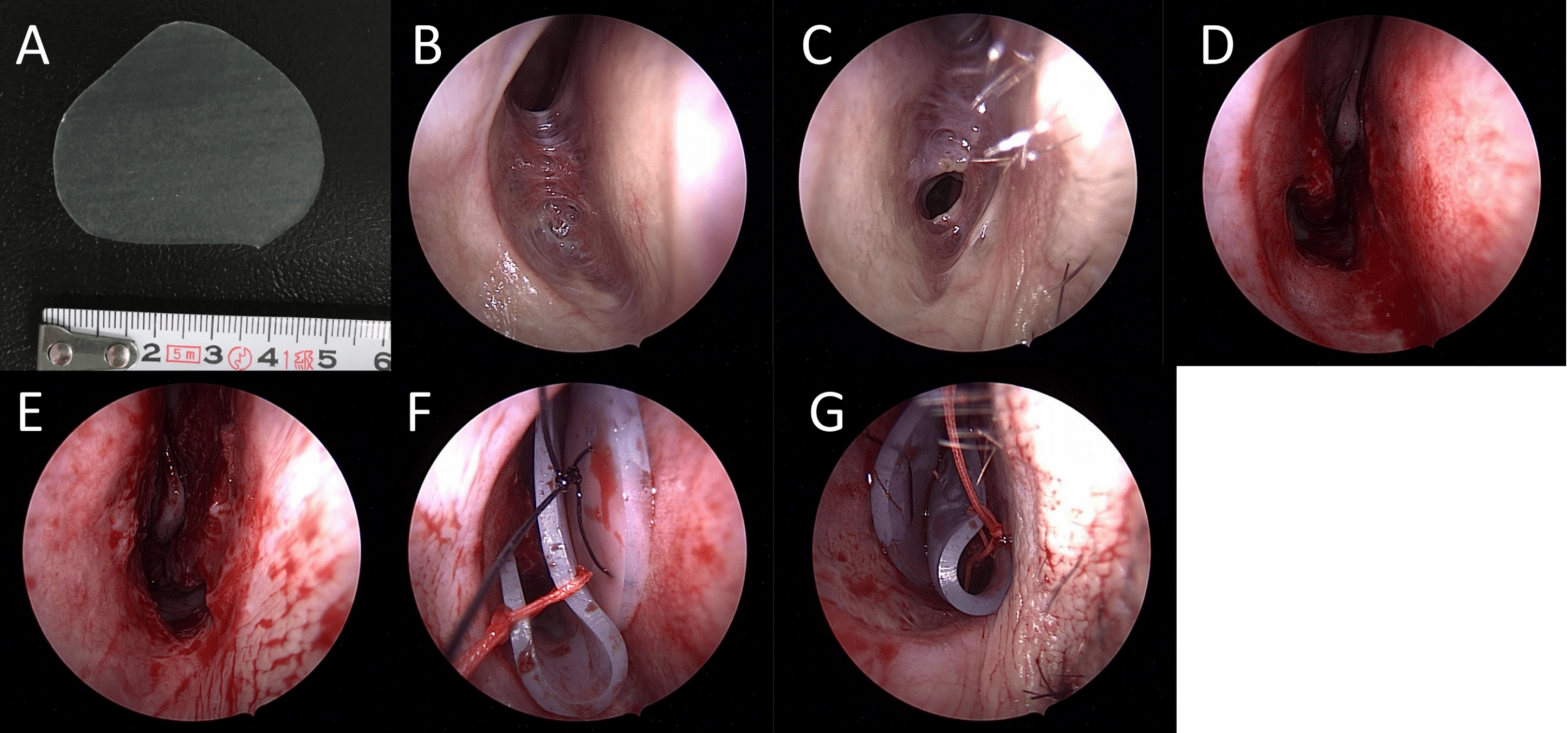
Recordings from the second surgery performed under general anesthesia. A) 1 mm silicone sheets shaped as shown. The scale bar represents 1 cm. B) Right nostril at the beginning of the surgery. C) Left nostril at the beginning of the surgery. D) Right nostril after adhesion division. E) Left nostril after adhesion division. F) Insertion of a complex of silicone sheets in the right nostril. G) Insertion of a complex of silicone sheets in the left nostril.

The cause of the adhesions was unclear. He did not have any congenital condition that could have led to the adhesions. We performed adhesion division in the outpatient clinic under local anesthesia on the same day. Due to severe bleeding caused by untreated hypertension, we were unable to divide the adhesions at the inferior meatus. A bent silicone sheet (Figure 2A) was placed in each nostril for one week, and the patient was prescribed Tranilast 300 mg daily. Amoxicillin 1500 mg and tranexamic acid 750 mg daily were also prescribed for seven days.

At two weeks post-operation, narrowing of the nasal airway and crust formation were noted. By six weeks, small adhesion had developed in both nostrils. As the patient did not experience nasal obstruction at that time, he was referred back to the clinic for further follow-up.

Two years after the initial surgery, the patient was re-referred to our department due to recurrent nasal obstruction. Upon examination, extensive adhesions, similar to the initial presentation, were observed (Figures 2B, 2C).

**Figure 3:**
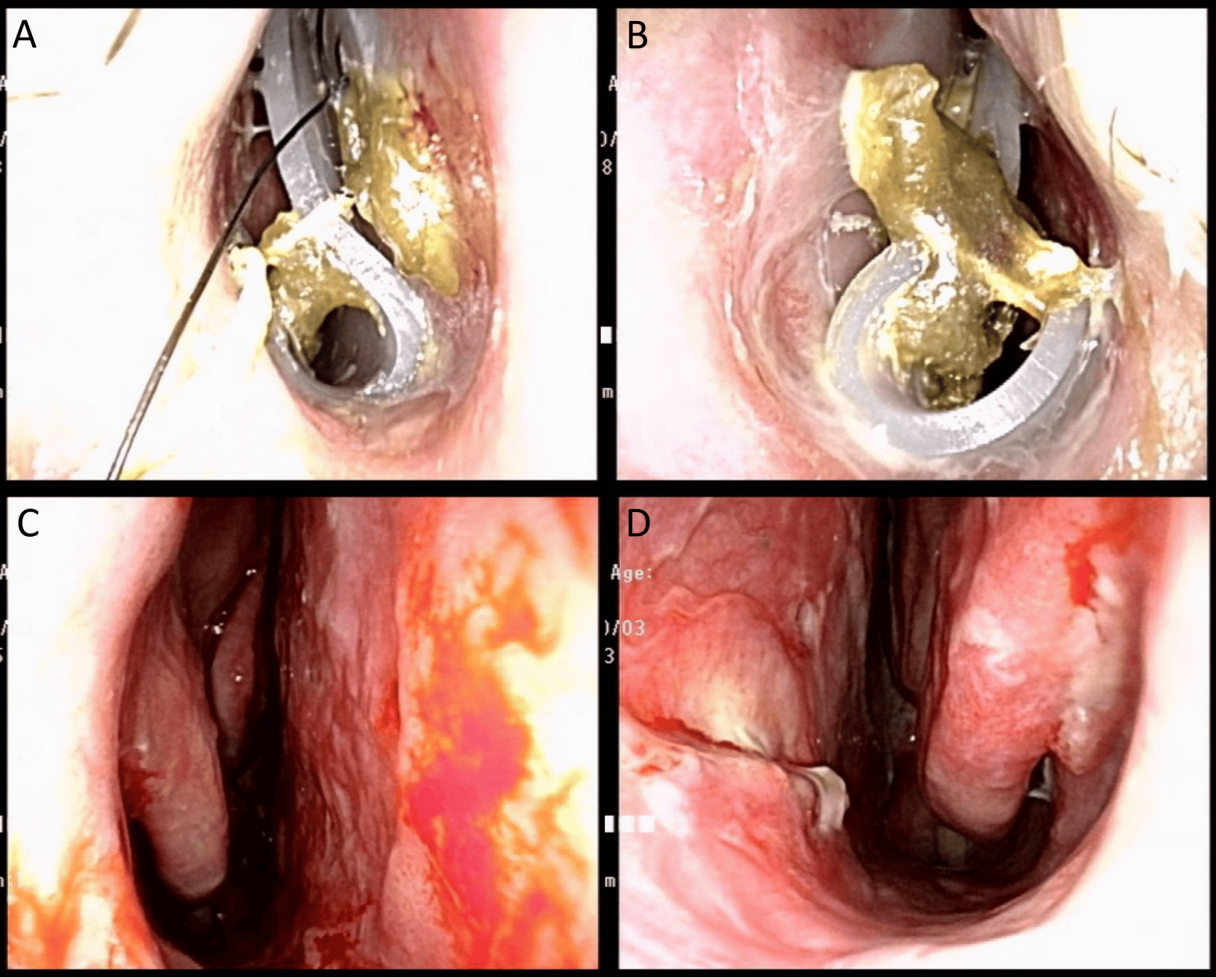
Recordings four weeks after the second operation. A) Right nostril. B) Left nostril. C) Right nostril after silicone sheet removal. D) Left nostril after silicone sheet removal.

To ensure complete remission, adhesion division was performed under general anesthesia (Figures 2D, 2E). The adhesion tissue was resected using cutting forceps. Two complexes of 1 mm silicone sheets (#0412, KOKEN CO., LTD. Japan) cut into the triangular shape (Figure 2A), each consisting of flat and bent components (fixed using 2-0 silk sutures), were inserted into both nostrils. A single 4-0 nylon suture was used to fix both complexes to the nasal septum, and they were maintained for four weeks (Figures 2F, 2G). Cefazolin 2 g was administered at the beginning of the surgery, and the same dose was given again six hours later. Due to a foul smell, cefaclor 750 mg daily was prescribed starting on post-operative day 18 for seven days. The silicone sheets were removed at the outpatient office four weeks after the operation (Figure 3).

**Figure 4:**
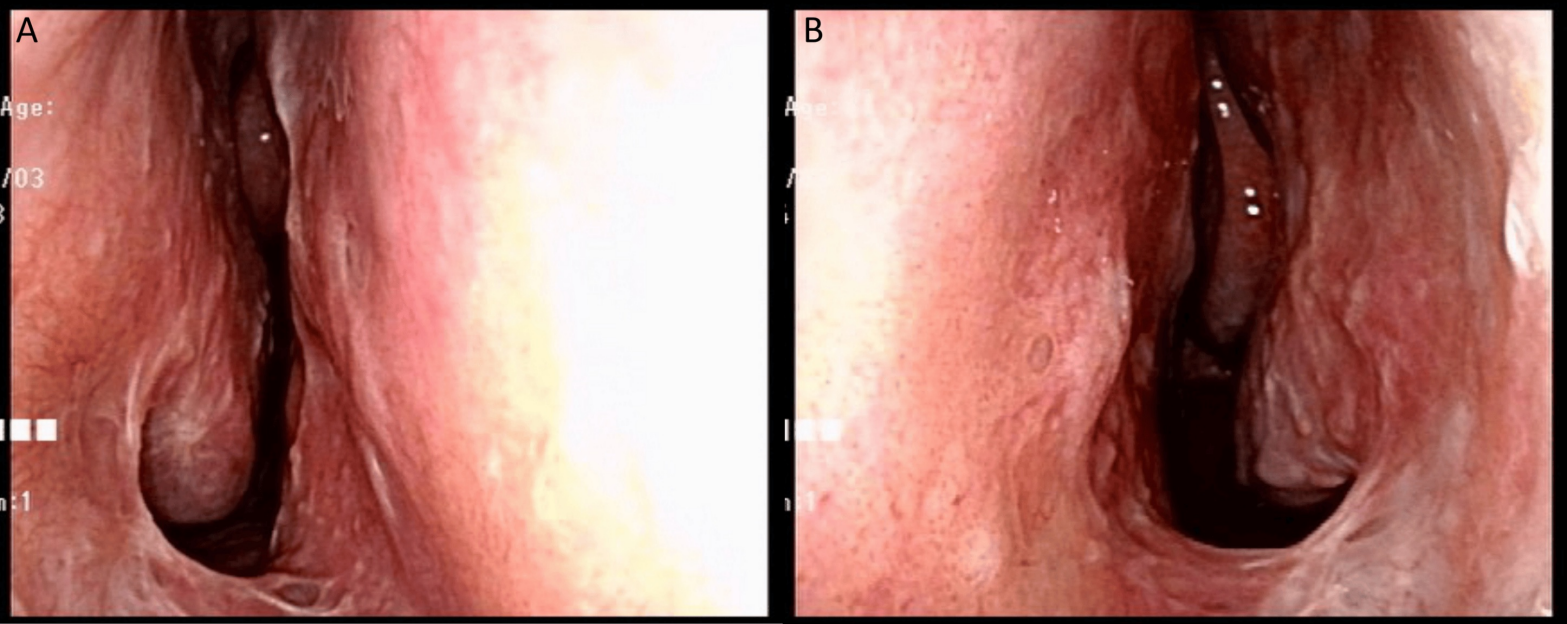
Recordings one year after the second operation. A) Right nostril. B) Left nostril.

Tranilast (300 mg daily) was administered for three months, and Fluticasone Furoate nasal spray (55 μg per nostril daily) was continued for five months. The nasal airway remained open for one-year post-procedure (Figure 4).

Written informed consent was obtained from the patient.

## Discussion

Nasal adhesions or synechiae in the anterior nasal cavity are rare, but the associated symptoms can be quite bothersome for patients. When adhesions are in the early stages, a simple procedure can often resolve them completely [5]. This approach is particularly effective for patients prone to adhesion formation, such as those undergoing radiation therapy for nasopharyngeal carcinoma, through close monitoring during treatment. However, treating severe adhesions can be challenging. Even with adhesion division and subsequent packing, recurrences are common. Our technique is straightforward, with silicone sheets placed to span both the common meatus and the inferior meatus.

Silicone sheets are commonly used for the splints for operated nasal septum after septoplasty [2,6]. Silicone sheets have a positive effect on the septal mucosal healing process after septoplasty [2]. By placing the silicone sheet, even where healthy mucosa does not exist, a fibrous membrane forms around the silicone sheet [7]. Silicone sheets have several merits over other packing materials or spacers. Removal of silicone sheets has little effect on bleeding, patient discomfort, and pain [6]. Silicone's smooth surface makes biofilm formation less likely than Merocel (polyvinyl acetal) [8]. Silicone is known for its high biocompatibility, making it an ideal material for medical implants. It is chemically inert, which means it does not easily react with bodily tissues. Multiple studies have demonstrated that silicone causes minimal inflammatory response when used in various medical applications [9]. This low level of tissue reaction reduces the likelihood of significant inflammation or immune responses, and it helps to prevent tissue assimilation or excessive scarring around the implant.

Silicone sheets are commonly used as splints for the nasal septum after septoplasty [2,6], and they positively influence the healing of the septal mucosa [2]. Even in areas lacking healthy mucosa, silicone sheets facilitate the formation of a fibrous membrane around them [7]. They offer several advantages over other packing materials or spacers: their removal causes minimal bleeding, discomfort, and pain for patients [6]. Additionally, the smooth surface of silicone sheets reduces the likelihood of biofilm formation compared to Merocel (polyvinyl acetal) [8]. Silicone’s high biocompatibility makes it an ideal material for medical implants. Being chemically inert, it does not easily interact with bodily tissues, resulting in minimal inflammatory responses [9]. Silicone sheets were sterilized by autoclaving at 135°C for 8 minutes. This low level of tissue reaction decreases the risk of significant inflammation or immune responses and helps to prevent tissue assimilation or excessive scarring around the material.

Our initial attempt was unsuccessful. We placed flat silicone sheets in both nostrils for one week, but because the procedure was performed under local anesthesia in an outpatient setting, we were unable to divide the adhesions at the inferior meatus. During the second attempt, after a complete recurrence of the adhesions, we opted for general anesthesia. This allowed for the complete removal of adhesions in both the common and inferior meatus. To maintain the airway, we placed a combination of silicone sheets-one flat and one bent-secured with sutures. By keeping the silicone sheets in place for four weeks, a tissue resembling mucosa likely formed over their surface. The airspace remained open for one year following the procedure. Although the silicone sheets are flexible, they may straighten and shift without adequate support, making suturing necessary. Cefaclor 750 mg daily was administered for 7 days midway through the 4-week silicon sheet insertion. At that time, a noticeable foul smell was present. Placing any foreign body for an extended period carries a risk of infection. There are limitations in this report, including that it is a single-patient case and the lack of long-term follow-up beyond one year. Accumulation of further cases is needed to clarify the effect of silicone sheets in the treatment of adhesions.

## Conclusions

Silicone is so far the best material to cover the raw surface of the nasal cavity. Being chemically inert, it does not easily interact with bodily tissues, resulting in minimal inflammatory responses. This low level of tissue reaction decreases the risk of significant inflammation or immune responses and helps to prevent tissue assimilation or excessive scarring around the material.

Silicone sheets secured with sutures are likely the optimal material for covering the raw surface after adhesion division. We used nylon, a monofilament material, to minimize the risk of infection, but had to administer Cefaclor to suppress the foul smell during the prolonged insertion of silicone sheets. Further accumulation of patient data is needed to verify the effectiveness of silicone sheets.
